# Identification of Key *TRIM* Genes Involved in Response to *Pseudomonas aeruginosa* or *Chlamydia* spp. Infections in Human Cell Lines and in Mouse Organs

**DOI:** 10.3390/ijms241713290

**Published:** 2023-08-27

**Authors:** Ekaterina Stepanenko, Natalia Bondareva, Anna Sheremet, Elena Fedina, Alexei Tikhomirov, Tatiana Gerasimova, Daniil Poberezhniy, Irina Makarova, Vyacheslav Tarantul, Nailya Zigangirova, Valentina Nenasheva

**Affiliations:** 1 Laboratory of Molecular Neurogenetics and Innate Immunity, National Research Centre “Kurchatov Institute”, Moscow 123182, Russia; katishsha@mail.ru (E.S.);; 2 Laboratory for Chlamydiosis, National Research Center for Epidemiology and Microbiology Named after N. F. Gamaleya, Russian Health Ministry, Moscow 123098, Russia; 3 Department of Chemistry and Technology of Biomedical Pharmaceuticals, D. Mendeleev University of Chemical Technology of Russia, Moscow 125047, Russia

**Keywords:** *TRIM* family genes, innate immune response, *Pseudomonas aeruginosa*, *Chlamydia* spp.

## Abstract

Bacterial infections represent an unsolved problem today since bacteria can evade antibiotics and suppress the host’s immune response. A family of TRIM proteins is known to play a role in antiviral defense. However, the data on the involvement of the corresponding genes in the antibacterial response are limited. Here, we used RT-qPCR to profile the transcript levels of *TRIM* genes, as well as interferons and inflammatory genes, in human cell lines (in vitro) and in mice (in vivo) after bacterial infections caused by *Pseudomonas aeruginosa* and *Chlamydia* spp. As a result, the genes were identified that are involved in the overall immune response and associated primarily with inflammation in human cells and in mouse organs when infected with both pathogens (*TRIM7*, *8*, *14*, *16*, *17*, *18*, *19*, *20*, *21*, *47*, *68*). *TRIMs* specific to the infection (*TRIM59* for *P. aeruginosa*, *TRIM67* for *Chlamydia* spp.) were revealed. Our findings can serve as a basis for further, more detailed studies on the mechanisms of the immune response to *P. aeruginosa* and *Chlamydia* spp. Studying the interaction between bacterial pathogens and the immune system contributes to the search for new ways to successfully fight bacterial infections.

## 1. Introduction

The duration and clinical outcome of a bacterial infection are determined not only by the characteristics of pathogenic bacteria, but also by their interaction with the innate immune system, which also makes a significant contribution. An important part of the immune system is the TRIM family, which includes about 80 proteins with a common structural N-terminal TRIpartite motif [1]. TRIMs are known to be actively involved in the antiviral response of innate immunity [2], while their role in the antibacterial response has not yet been studied enough. In recent years, there has been evidence on the involvement of TRIMs in the functioning of the innate immune system during bacterial infection [3,4,5,6]. Chen et al. (2018) showed that the expression of twenty *TRIM* genes was decreased in the peripheral blood cells of active tuberculosis patients infected with *Mycobacterium tuberculosis* in comparison with healthy individuals [3]. TRIM27 and TRIM22 were demonstrated to inhibit the survival of *M. tuberculosis* [4,5], whereas the knockout of *TRIM14* in macrophages restricted the reproduction of *M. tuberculosis* [7]. TRIM21, TRIM56, and TRIM65 regulated the innate immune response during *Salmonella enterica* invasion [8,9]. The deficiency of *TRIM32* was shown to significantly reduce bacteremia and pro-inflammatory cytokine production after *Streptococcus suis* infection [10]. Therefore, the available data demonstrate that TRIMs are able to both suppress bacterial infections or to serve as drivers of bacterial invasion.

The two types of pathogenic bacteria we have chosen for our study (*Pseudomonas aeruginosa* and *Chlamydia* spp.) are of particular interest, since the infections they cause are widespread in the world, especially among people with weakened immune systems [11,12]. They differ in the way they interact with the cell and refer to extracellular (*Pseudomonas aeruginosa*) and obligate intracellular (*Chlamydia* spp.) pathogens. Both bacteria are able to induce and constitutively maintain an inflammatory response, as well as bypass the host’s immune defense mechanisms. *P. aeruginosa* is an extremely problematic causative agent of respiratory and urinary diseases, with a whole arsenal of virulence factors aimed at suppressing the immune response.

We hypothesized that *TRIMs* could be actively involved in the immune system’s response to these infections. In our work, we studied the expression profiles of *TRIM* genes upon *P. aeruginosa* and *Chlamydia* spp. infections in human cell lines and in mice and found both similarities and differences. The data obtained allowed us to make an assumption about the general mechanisms of the *TRIM* genes’ work during these infections, as well as propose specific *TRIM* genes that may be involved in the antibacterial response.

## 2. Results

In our study, we examined both the local and systemic responses of *TRIM* genes to *P. aeruginosa* or *Chlamydia* spp. infections in models in vitro (human cell lines) and in vivo (DBA/2 mice), compared with non-infected controls, using RT-qPCR analysis. We considered the A549 cell line (human lung carcinoma), PC-3 cells (human prostatic adenocarcinoma), or mouse lungs as models of the primary sites of infection. The monocytic U937 cell line served as a model of the systemic immune response, and the axillary lymph nodes represented the site of adaptive immunity. The expression of several *TRIM* genes independently of the infections was below the level of detection in the selected cell lines or in mouse organs. In addition, not all homologue genes were found in both humans and mice. Therefore, the data were not presented for all 75 genes in the *TRIM* family. The expression was observed for 64, 51, and 53 *TRIM* genes in A549, U937, and PC-3 cell lines, respectively. Specifically, 57 *TRIM* genes were analyzed in the lungs and 52 *TRIM* genes in the lymph nodes of DBA/2 mice after infections compared to intact animals. We considered the gene up-regulated or down-regulated when its expression changed by more than 1.5 times.

### 2.1. The Expression Profiles of TRIM Genes in Human A549 and U937 Cell Lines and in DBA/2 Mouse Organs after P. aeruginosa Infection

**Figure 1:**
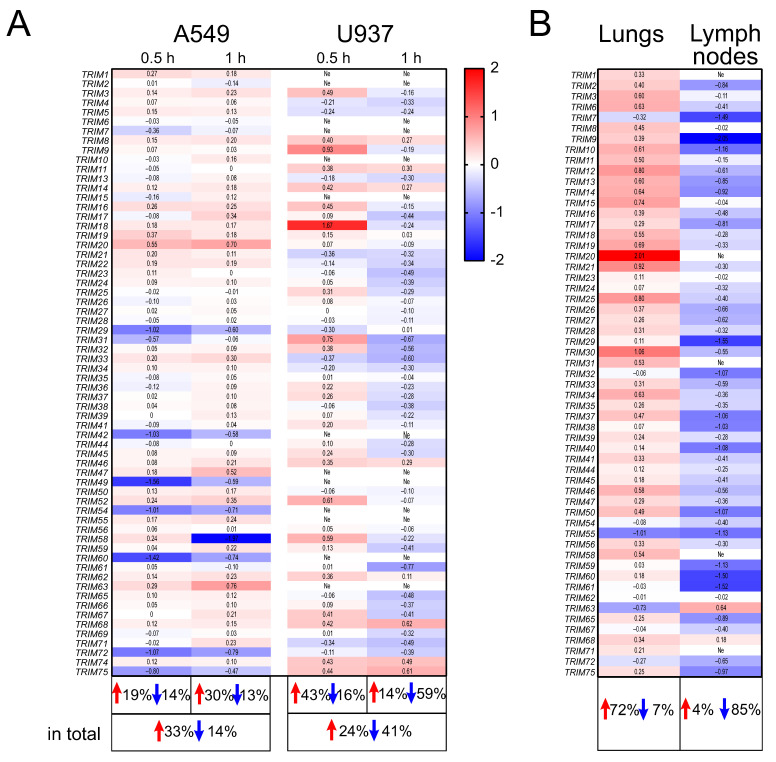
The changes in *TRIM* gene expression in human cell lines and in mouse organs after *P. aeruginosa* infection. Heatmaps of the *TRIM* gene expression in (**A**) A549 and U937 cell lines 0.5 h and 1 h p.i., and (**B**) mouse lungs and lymph nodes 48 h p.i. Data are presented as lgFC (gene expression p.i./gene expression in the control cells or organs). Red color: up-regulation; blue color: down-regulation. The percentage is indicated for genes with FC > 1.5 (red arrows: up-regulated genes; blue arrows: down-regulated genes). Ne: no expression.

*Pseudomonas aeruginosa* causes different severe infections, including pneumonia. We chose the A549 cells as the lung cell model and the monocytic U937 cell line for studying the non-specific immune response. To gain insights into the early response to *P. aeruginosa*, the analysis was conducted at 0.5 h and 1 h post infection (p.i.). We observed a massive increase in the *TRIM* gene expression in the A549 cell line upon *P. aeruginosa* infection (33% of up-regulated genes vs. 14% of the down-regulated genes) (Figure 1A). At the same time, in the U937 cell line, *P. aeruginosa* caused an increase in the expression of 43% of the *TRIM* genes 0.5 h p.i. and a decrease in the expression of 59% of the *TRIM* genes 1 h p.i. (Figure 1A). Presumably, the initial activation of certain *TRIM* genes was followed by massive suppression in the monocyte cell line.

Further, we found out what happened to the *TRIM* gene expression in vivo in mice after infection with *P. aeruginosa*. The expression level of most of the *TRIM* genes tended to increase in the lungs (72%) and decrease in the lymph nodes (85%) of mice infected with *P. aeruginosa* in comparison with intact (control) mice (Figure 1B). Therefore, up-regulation of the *TRIM* genes was noticed both in human A549 cells and in mouse lungs after *P. aeruginosa* (PA) infection (a pairwise comparison of the expression profiles showed no significant difference: PA lungs vs. PA A549 at 1 h p.i., *p* = 0.498), while the expression of many *TRIMs* was decreased in human U937 cells at 1 h p.i. and in mouse lymph nodes at 48 h p.i. (PA lymph nodes vs. PA U937, *p* = 0.944).

Since not all the changes in the *TRIM* gene expression were significant, we chose for further analysis those for which *p* < 0.05 (Table 1 and Table 2).

We noted similarities between the in vitro and in vivo *TRIM* gene transcription changes in both A549 vs. mouse lungs and U937 vs. mouse lymph nodes cases. As for mouse lung and human lung epithelial cells, *TRIM16*, *17*, *18*, *19*, *20*, and *21* were up-regulated in both models after *P. aeruginosa* infection, and *TRIM7* expression was decreased in both models, as well as in mouse lymph nodes. The expression of *TRIM12*, *13*, *14*, *17*, *21*, *26*, *27*, and *56* genes appeared to be increased in the lungs and decreased in the lymph nodes. Interestingly, *TRIM63* was up-regulated in A549 cells; however, its homologue was down-regulated in mouse lungs. *TRIM59* and *65* appeared to be down-regulated in the monocytes and mouse lymph nodes.

### 2.2. The Expression Profiles of TRIM Genes in Human A549, U937, and PC-3 Cell Lines and in DBA/2 Mouse Organs after Chlamydia spp. Infection

To investigate the expression of *TRIMs* in *Chlamydia* spp. infection, we chose three representatives of the pathogen. For human cell lines, we used *C. pneumoniae*, which is an etiological agent of up to 20% of community-acquired atypical pneumonia, bronchitis, and upper respiratory tract infections, and *C. trachomatis*, the most common bacterial pathogen that causes acute and chronic infections of the reproductive organs, leading to infertility, pregnancy pathology, and infections in newborns. For the mouse infection, we used *C. muridarum*, a murine obligate intracellular pathogen that is widely included in mouse models of chlamydial infections of the respiratory and genital tract [13]. Since *Chlamydia* spp. infection progresses more slowly compared to *P. aeruginosa* invasion, we chose longer periods of time until the moment of analysis: 4 and 8 h p.i. in cell lines and 72 h p.i. in mice.

**Figure 2:**
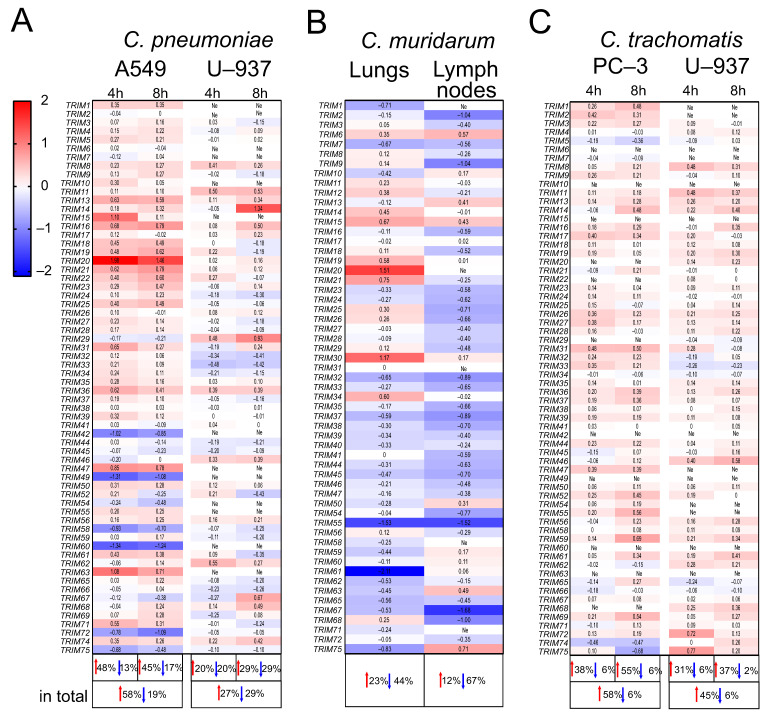
The changes in *TRIM* gene expression in human cell lines and in mouse organs after *Chlamydia* spp. infection. Heatmaps of the expression of *TRIM* genes in (**A**) A549 and U937 cell lines 4 and 8 h p.i. with *C. pneumoniae*; (**B**) mouse lungs and lymph nodes 72 h p.i. with *C. muridarum*; and (**C**) PC-3 and U937 cell lines 4 and 8 h p.i. with *C. trachomatis*. Data are presented as lgFC (gene expression p.i./gene expression in the control cells or organs). Red color: up-regulation; blue color: down-regulation. The percentage is indicated for genes with FC > 1.5 (red arrows: up-regulated genes; blue arrows: down-regulated genes). Ne: no expression.

In the A549 cell line, we observed a massive increase in *TRIM* gene expression (58% of the examined genes) upon *C. pneumoniae* infection; likewise, in U937, the number of genes with increased or decreased expression turned out to be approximately the same (in total, 27% and 29% of the genes analyzed, respectively) (Figure 2A). Only 23% of the genes were up-regulated after pulmonary *C. muridarum* infection in mouse lungs, whereas the expression of 44% of genes was decreased. Also, a massive decrease in *TRIM* gene expression in the lymph nodes was observed after *C. muridarum* infection (67%) (Figure 2B). Notably, *TRIM8, 14, 15, 19, 20, 21, 56*, and *68* were up-regulated in both the A549 cells and mouse lungs after pulmonary *Chlamydia* spp. infections (Table 1). Only *TRIM67* was down-regulated in both models (Table 2). Interestingly, there were no compatible changes in U937 and the lymph nodes after pulmonary *Chlamydia* spp. infections, as was detected after *P. aeruginosa* infection (Table 1 and Table 2). As for *TRIM8*, its expression was increased in the U937 cells and decreased in the mouse lymph nodes (Table 1 and Table 2). It should be pointed out that *TRIM7* was down-regulated in both the lungs and lymph nodes after infection with *C. muridarum* and *P. aeruginosa*.

Next, we considered the prostatic adenocarcinoma cell line PC-3 as an adequate model for *C. trachomatis* infection, which usually causes urogenital pathology [14]. We observed an increase in the expression of 58% of the *TRIM* genes in infected PC-3 (Figure 2C), as in A549 upon *C. pneumoniae* infection (CT PC-3 vs. CP A549, 4 h p.i., *p* = 0.973, 8 h p.i., *p* = 0.902). In U937, the expression of a large part of the *TRIM* genes was also increased (45% of genes) (Figure 2C), in contrast to gene expression in *C. pneumoniae* infection.

Overall, here are some promising results generalized for both infections and both models (Table 1 and Table 2). It should be noted that the pulmonary infections caused by *P. aeruginosa,* as well as *C. pneumoniae,* led to increased expression of a large pool of the same *TRIM* genes both in the A549 cell line and in mouse lungs (Table 1) (a pairwise comparison of the expression profiles showed no significant difference: PA lungs vs. PA A549 1 h p.i., *p* = 0.498; PA A549 0.5 h p.i. vs. CP A549 4 h p.i., *p* = 0.213; PA A549 0.5 h p.i. vs. CP A549 8 h p.i., *p* = 0.454; PA A549 1 h p.i. vs. CP A549 4 h p.i., *p* = 0.8; PA A549 1 h p.i. vs. CP A549 8 h p.i., *p* = 0.958). On the contrary, in the mouse lymph nodes after both infections, many of the same *TRIM* genes were down-regulated, demonstrating similar mechanisms of the immune response. At the same time, some of the genes were unique for each infection and cell type (highlighted in bold in Table 1 and Table 2).

Summarizing, several genes were up-regulated in the cell lines that we considered to be a model of the primary site of infection: *TRIM16, 47* in A549 and PC-3 after *P. aeruginosa* and *Chlamydia* spp. infections; and *TRIM18*, *19*, *20*, *21, 63* in A549 cells in response to both pulmonary infections. Several of these genes, namely *TRIM16*, *17*, *18*, *19*, *20*, *21*, were also up-regulated in mouse lungs after *P. aeruginosa*, and three of them, *TRIM19*, *20*, *21*, after both pulmonary infections (*P. aeruginosa* and *C. pneumoniae*) (Table 1). We consider these genes to participate in the early non-specific immune response, which is activated at the point of contact with various bacteria. *TRIM8*, *14,* and *68* were increased in U937 cells in response to all the pathogens examined, as well as in mouse lungs upon both infections, and in A549 after *C. pneumoniae* infection (Table 1). *TRIM7* and *63* were down-regulated in mouse lungs upon *P. aeruginosa* and *C. muridarum* infections. Most interestingly, *TRIM7* expression decreased in the lungs and in the lymph nodes after both infections. *TRIM65* was down-regulated in U937 cells after *P. aeruginosa* and *C. trachomatis* infection, in mouse lungs and lymph nodes after *C. muridarum* infection, and in lymph nodes upon *P. aeruginosa* infection (Table 2). In addition, we found that the dynamics of *TRIM63* expression during both bacterial infections in mice (in the lungs, Table 2) was opposite to that in humans (in A549 cells, Table 1), which means that the mouse model is not suitable for studying the role of *TRIM63* in human bacterial-caused diseases.

In order to determine the possible pathway of bacteria’s influence on the *TRIM* genes, we compared our results with those in recent studies where the expression of *TRIM* genes upon Toll-like receptor (TLR) activation in THP1-derived macrophages [15], or after interferon (IFN) type I or II stimulation in primary monocyte-derived macrophages, or peripheral blood lymphocytes [16] was analyzed. We found that the majority of the *TRIMs* that were activated after *P. aeruginosa* or *Chlamydia* spp. infections (*TRIM5*, *13*, *14*, *15*, *18*, *19*, *20*, *21*, *22*, *25*, *26*, *31*, *35*, *36*, *37*, *50*, *55*, *56*, *61*, *63*, *65*, *69,* and *71*) were up-regulated following TLR stimulation and/or by IFNs (Table S2), while *TRIM59* and *TRIM66* were down-regulated under these conditions and upon *P. aeruginosa* infection (Table S2).

Likewise, we found that several *TRIMs* (*TRIM 13*, *14*, *15*, *18*, *19*, *20*, *21*, *25*, *26*, and *56*) activated in *P. aeruginosa* or *C. muridarum* infections were also up-regulated following TLR activation and/or stimulation with IFNs (Table S3). Notably, *TRIM8*, *16*, *17*, and *27* transcription was increased and the expression of *TRIM65* and *67* was decreased in the response to various pathogens in different human cell lines and in mouse lungs as well (Tables S2 and S3), suggesting a universal antibacterial mechanism for these genes in mice and humans.

Additionally, we performed bioinformatic analysis of the RNA-seq data on transcriptomes of mouse lung after *P. aeruginosa* (24 h p.i.) and *C. muridarum* (7 days p.i.) infections published by Ebenezer et al. (2019) [17] and Virok et al. (2019) [18] and demonstrated the same trends in the expression of *TRIM* genes, taking into account the difference in the time of sampling (Table S3).

### 2.3. Western Blot Protein Assay of Several TRIMs of Interest in the U937 Cell Line

**Figure 3:**
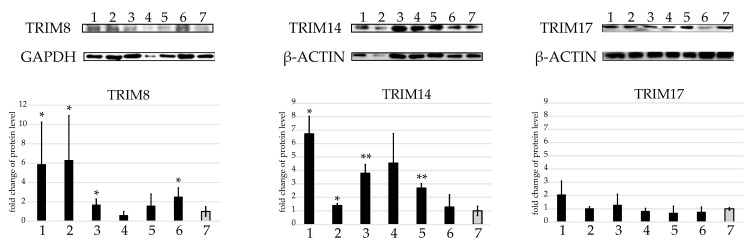
TRIM8, TRIM14, and TRIM17 protein level changes in the U937 cell line after *P. aeruginosa* and *Chlamydia* spp. infections. Western blot analysis (and quantification below, n = 3) of TRIM8, TRIM14, and TRIM17 protein levels in the U937 cell line after infections: (1) 4 h p.i. with *C. trachomatis*; (2) 8 h p.i. with *C. trachomatis*; (3) 4 h p.i. with *C. pneumoniae*; (4) 8 h p.i. with *C. pneumoniae*; (5) 0.5 h p.i. with *P. aeruginosa*; (6) 1 h p.i. with *P. aeruginosa*; (7) intact cells. GAPDH or β-ACTIN were used as references for data normalization. * *p* < 0.05, ** *p* < 0.01.

Next, we selected three *TRIM* genes, namely *TRIM8*, *14*, and *17*, to study changes in the expression of the corresponding proteins in cell line U937 after infection with *P. aeruginosa*, *C. pneumoniae*, and *C. trachomatis*. Western blot analysis showed changes in TRIM8 and TRIM14 protein expression, confirming the involvement of the proteins in the immune response to the studied bacterial infections (Figure 3). Expression of the TRIM17 protein remained practically unchanged, as was its transcription. (Figure 3).

### 2.4. The Expression Profiles of IFNs and Inflammatory Genes in Human A549, U937, and PC-3 Cell Lines, and in DBA/2 Mouse Organs after P. aeruginosa and Chlamydia spp. Infections

**Figure 4:**
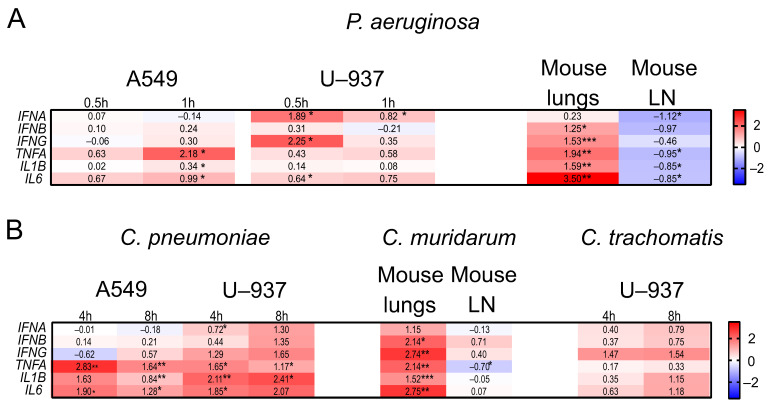
Analysis of the expression of genes encoding IFNs and pro-inflammatory proteins in human cell lines and mouse organs after *P. aeruginosa* and *Chlamydia* spp. infections. Heatmaps of the immune gene expression in the A549 and U937 cell lines and mouse lungs and lymph nodes after (**A**) *P. aeruginosa* and (**B**) *Chlamydia* spp. infections. Data are presented as lgFC (gene expression after infection/gene expression in the control cells or organs). Red color: up-regulation; blue color: down-regulation. * *p* < 0.05, ** *p* < 0.01, *** *p* < 0.001.

Since *TRIM* genes are known to be interferon-stimulated genes (ISGs) and also to participate in the inflammatory response, we analyzed type I IFNs (*IFNA*, *B*), a type II IFN (*IFNG*), and pro-inflammatory gene (*TNFA*, *IL1B*, and *IL6*) expression using RT-qPCR, both in vitro and in vivo (Figure 4).

The IFN system was mostly involved in response to both types of infection in mouse lungs and in U937 cells, while in A549, the transcription of IFNs remained unchanged or slightly increased (the expression of *IFNG* was even decreased at 4 h p.i. with *C. pneumoniae*) (Figure 4). Importantly, *TNFA*, *IL1B*, and *IL6* were up-regulated in response to the bacterial infections in both epithelial A549 and monocyte U937 cells, as well as in vivo in the lungs (Figure 4). Additionally, a significant increase in the expression of pro-inflammatory genes (*TNFA*, *IL1B*, and *IL6*) in PC-3 cells infected with *C. trachomatis* was reported earlier [14], which was in line with our observations.

However, in mouse lymph nodes, genes encoding both the *IFNs* and pro-inflammatory cytokines were suppressed after *P. aeruginosa* infection. *TNFA*, *IL1B*, and *IL6* gene expression dropped when mice were infected with *C. muridarum*, while *IFNB* and *IFNG* expression slightly increased (Figure 4). Given that the lymph nodes are responsible for adaptive immunity that becomes detectable within the lymph nodes after 5 days p.i. [19,20], we assume that the *IFN* and pro-inflammatory gene programs were not yet activated in the lymph nodes in our models of the early stages of acute infections.

Thus, it can be supposed that the described changes in the expression of the *TRIM* genes after infection may be due to the TLRs activation by bacterial molecular patterns and cannot be explained by the activation of only interferon signaling.

## 3. Discussion

Throughout life, we constantly encounter infections caused by various pathogens. The formation of an effectively protective immune response depends on the functioning of many adapter molecules involved in the transmission, as well as the amplification or attenuation, of the signal from receptors, which meet pathogens, to the transcription factors in eukaryotic cells. Among currently known adapter molecules, proteins from the TRIM family are of particular interest since they are shown to be actively involved in the antiviral response of innate immunity [2,21]. However, there is still very little information regarding the role of TRIM proteins in the antibacterial response. In our opinion, new findings in this field will contribute to a more comprehensive understanding of intracellular signaling cascades involved in the immune response.

*P. aeruginosa* and *Chlamydia* spp. are pathogens that can successfully fight the immune system [11,12], due to the high level of antibiotic resistance and tolerance to the action of many antibacterial drugs, inherent in these pathogens. This determines the need for fundamental studies on the molecular mechanisms of the interaction of these pathogens with the host’s innate immune system and the search for new approaches to combat diseases caused by these extremely problematic pathogens.

**Figure 5:**
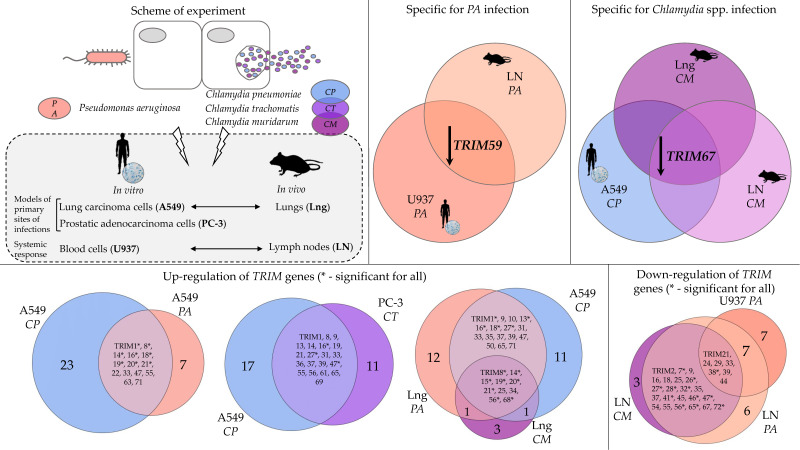
Observed similarities and differences in *TRIM* gene expression in human cell lines and in mouse organs upon two different types of infection caused by *P. aeruginosa* and *Chlamydia* spp.

Our study showed similarities and differences in expression for a number of *TRIM* genes after *P. aeruginosa* and *Chlamydia* spp. Infections, both in vitro (human cell lines A549, U937, and PC-3) and in vivo (lungs and lymph nodes of mice) (Figure 5). Activation and deactivation of the *TRIM* genes occurred quite synchronously, depending on the types of cells and pathogens. Namely, two different pathogens, *P. aeruginosa* and *C. pneumoniae*, caused an increase in *TRIM* expression in A549 cells and in mouse lungs. We assumed that such a correspondence might be due to the fact that the lungs were a primary focus for both infections in vivo and that A549 cells are related to lung tissue due to the cells’ nature. In the same way, mass up-regulation of the *TRIM* genes was found in the prostatic adenocarcinoma cell line PC-3 after *C. trachomatis* infection, which causes damage to the genital tract. Thus, we suggest that the expression of *TRIM* genes apparently depends on whether the cells belong to the primary site of infection or not.

Strikingly, we also observed the massive suppression of *TRIM* gene expression in the lymph nodes after infections with *P. aeruginosa* and *C. muridarum.* On the one hand, that could be explained by the fact that during acute infection, activation of the adaptive immunity in the lymph nodes is delayed. On the other hand, such a suppression of *TRIMs* and even of non-specific pro-inflammatory genes (*TNFA*, *IL1B*, and *IL6*) might be a result of the active influence of pathogens on the host’s immune system. It should be mentioned that several *TRIM* genes, whose expression was decreased in the lymph nodes after bacterial infections, were previously reported to take part in T-cell signaling (*TRIM21*, *27*, *28*, *32*, *33*) [22]. It is possible that bacteria, counteracting the immune defense, suppress the activation of this group of *TRIM* genes.

Additionally, our data on the down-regulation of *TRIM17*, *21*, *27*, *32*, *35*, *45*, *46*, *47*, *56*, and *65* in the mouse lymph nodes correlate with those obtained by Chen et al. [3] in the blood of tuberculosis patients and/or in macrophages after infection with *Mycobacterium smegmatis* [3].

Activation of the pro-inflammatory response is often the result of stimulation of TLRs, which are the main receptors in the innate immune system and are able to recognize pathogens of different natures depending on the type of receptor. There is evidence that *P. aeruginosa-* or *Chlamydia*-derived products are ligands for different TLRs [23,24,25]. TLR4 can specifically recognize gram-negative bacterial LPS and use both MyD88 and TRIF adaptor proteins, leading to the activation of genes encoding pro-inflammatory cytokines and interferons [26]. At the same time, many *TRIM* genes are IFN-inducible in the response to infections [16,27,28] and, in turn, can activate IFN production [2]. Our comparison of the *TRIM* gene expression changes upon *P. aeruginosa* or *Chlamydia* spp. infections with known data concerning the regulation of *TRIM* genes under stimulation of TLRs [15] or treatment by IFNs [16] revealed similar activation of many *TRIM* genes (*TRIM5*, *6*, *10*, *13*, *14*, *15*, *18*, *19*, *20*, *21*, *22*, *25*, *26*, *31*, *34*, *35*, *36*, *37*, *50*, *55*, *61*, *63*, *65*, *69*, *71* (in A549), *56*, *58* (in mouse lungs)) (Table S2 and S3). The expression of other *TRIM* genes was reduced in our study (mainly in the lymph nodes of mice), which was consistent with the corresponding data on monocyte derived macrophages or peripheral blood lymphocytes under IFN treatment [16], or under TLR stimulation [15] (*TRIM28*, *32*, *37*, *41*, *55* (in lungs), *59*, *61*, *66* (in U937) (Tables S2 and S3).

Our analysis, however, showed that although the *IFNs* were up-regulated in infected U937 monocytes and in the lungs of mice, they remained unchanged or only slightly increased in A549 cells upon both infections and were dramatically down-regulated in mouse lymph nodes under *P. aeruginosa* infection, suggesting their rather limited role in the bacterial infections we studied. The known literature data showing the involvement of many *TRIM* genes in inflammation [7,27,29,30,31,32,33,34,35,36,37,38] is supported by our data. Pro-inflammatory genes were found to be activated in all human cell lines, as well as in mouse lungs, after both pathogen infections. The exception was only mouse lymph nodes, especially after *P. aeruginosa* infection, where their expression dropped, which was also observed for the *IFNs* and for the majority of *TRIM* genes, as mentioned above. Interestingly, one of these genes with decreased expression is *TRIM72*, which was demonstrated to promote *P. aeruginosa*-induced inflammation in mouse lungs [39]. This gene was shown to act via the complement receptor (CR) in the Ig superfamily (CRIg) in alveolar macrophages [39]. This fact indicates the existence of various mechanisms for the participation of *TRIM* genes in the immune response to bacterial infections, not only through TLRs but also through other receptors.

It should be noted that the expression of the *TRIM63* gene changed in the opposite way in human cells and in mouse lungs. Therefore, caution should be exercised when extrapolating mouse data to humans.

Summarizing, our data show that bacterial pathogens directly influence signaling mechanisms of innate immunity, including a network of *TRIM* family genes (Figure 5). For the first time, we have compared the effects of two different pathogens in human cell lines (model system) and in animals (organism level) on the expression of *TRIM* family genes, as well as genes encoding interferons and pro-inflammatory factors. Among the identified genes, there are a number of *TRIM* genes that are involved in the overall immune response, both in vitro in human cells and in vivo in mouse organs, when infected with two different types of pathogens (*TRIM8*, *14*, *16*, *17*, *18*, *19*, *20*, *21*, *47*, *68*). Along with this, *TRIM* genes specific for both the pathogen and the organism were found (e.g., *TRIM59* upon *P. aeruginosa* infection in human U937 and mouse lymph nodes; *TRIM67* in mouse lungs and lymph nodes, as well as in A549 after *Chlamydia* spp. infections). The *TRIM7* gene was the only gene whose expression was decreased in mouse lungs and lymph nodes after both infections and in A549 after *P. aeruginosa* infection. Further study on the role of the *TRIM* genes, identified in our work, involved in the immune response to *P. aeruginosa* and *Chlamydia* spp. will allow us to determine, in more detail, the mechanisms of the formation of the antibacterial immune response to these pathogens.

## 4. Materials and Methods

### 4.1. Cell Lines

A549, a human lung carcinoma epithelial cell line (ATCC CRM-CCL-185); U937, a monocyte-like cell line (ATCC CRL-1593.2); and PC-3, a human prostatic adenocarcinoma cell line (ATCC CRL-1435), were cultured in DMEM high glucose (Gibco, Waltham, MA, USA) supplemented with 10% heat-inactivated fetal bovine serum (FBS) (Gibco, Waltham, MA, USA) at 37 °C in 5% CO_2_. All the cell lines were routinely tested for mycoplasma contamination.

### 4.2. Pathogens

*C. trachomatis* L2/434/Bu (ATCC VR 902B), *C. pneumoniae* TWAR strain TW-183 (ATCC VR-2282), and *C. muridarum* strain Nigg (ATCC VR-123) were used. *P. aeruginosa* clinical isolate KB-6/6/2014 was excreted from the bronchoalveolar lavage of patients from a Moscow hospital [40].

An isolation technique for *C. trachomatis*, *C. muridarum*, and *C. pneumoniae* using McCoy cells (ATCC^®^ CRL-1696™) was described in [41]. Elementary bodies (EB) were purified according to Miyashita and Matsumoto [42] in a Renografin gradient, suspended in SPG, and stored at −70 °C.

For measuring *C. trachomatis* and *C. pneumoniae* infectivity, confluent daily A549, PC-3 cell monolayers, or U-937 suspension cultures were infected with 10-fold dilutions of suspension of purified EB in 24-well plates (Corning Inc., Corning NY, USA), with 12 mm round cover glasses (Menzel, Berlin, Germany). After 48 h of incubation (5% CO_2_, 37 °C), the glasses were washed with 0.1 mM PBS, dried in air, and fixed in cold acetone for 15 min at room temperature (RT). The U-937 cells were precipitated by centrifugation for 10 min at 500 rpm, washed with 0.1 mM PBS, and fixed with ice-cold acetone for 15 min at RT.

The preparations were stained with monoclonal FITC-labeled antibodies for the species-specific protein antigen of *C. trachomatis* (Bio-Rad, Hercules, CA, USA) and for the genus-specific antigen of bacterial lipopolysaccharide to detect the antigen of *C. pneumoniae* (CABT-RM310, Creative Diagnostics, Shirley, NY, USA). The monolayer was examined by luminescent microscopy in a Nikon Eclipse Ni-U luminescent microscope (eyepieces 1.3, lens ×40). The percentage of infected cells in 30 independent visual fields was calculated, and the CFU per 1 mL was determined [43].

The 10-fold dilutions of a suspension from a night culture of *P. aeruginosa* KB-6 grown in LB broth were carried out, followed by seeding on cetrimide agar. Cultures were cultivated for 24 h at 37 °C. The count of the grown colonies of *P. aeruginosa* was carried out, and the CFU/mL was determined [40].

### 4.3. Infection of the Cell Lines

We determined the required multiplicity of infections: *C. trachomatis*—5 MOI, *C. pneumoniae*—10 MOI, *Pseudomonas aeruginosa*—5 MOI. After the addition of the infectious material, the culture plates were centrifuged at 3000 rpm for 60 min at 25 °C, and the cells were incubated at 5% CO_2_ at 37 °C. Further, the cell monolayer was washed with PBS and then lysed with TRIzol (Thermo Fisher Scientific, Waltham, MA, USA). Cell lysates were collected at 4 and 8 h after *Chlamydia* spp. infection, and at 0.5 and 1 h after *P. aeruginosa* infection in two duplicate experiments. The samples were stored at −70 °C.

### 4.4. Mouse Infection

The study was conducted in compliance with the Guide for the Care and Use of Laboratory Animals (NIH Publication #85–23, revised 1996) and with the recommendations in the national guidelines and was approved by the Gamaleya National Research Center Animal Care Committee (protocol #19, 2 July 2020).

Four-to-five-week-old female DBA/2 mice were randomly divided into 3 groups: intact mice (n = 10), *C. muridarum* pneumonia (n = 10), and *P. aeruginosa* pneumonia (n = 20). As we found previously, the *C. muridarum* Nigg strain [44] caused pneumonia in mice with a 5 × 10*^5^* CFU dose/animal and the *P. aeruginosa* clinical isolate KB6 caused pneumonia in mice with a dose of 1.1 × 10^7^ CFU per animal [40].

The mice were anesthetized with inhalational diethyl ether (Ecos-1, Moscow, Russia) and injected intranasally with 40 µL per mouse via saline containing 5 × 10*^5^* CFU of the *C. muridarum* Nigg strain, or 10^7^ CFU of *Pseudomonas aeruginosa*. After infection, the animals were monitored twice a day. During the accumulation of the pathogen in the lungs and the development of pneumonia, the condition of the mice worsened: food refusal; sticky hair; rapid, shallow breathing; and low activity appeared. On the second (*P. aeruginosa*) or third (*C. muridarum*) day after infection, the animals were subjected to euthanasia and subsequent autopsy. The lungs and axillary lymph nodes were taken from the mice in all the groups. The organs were treated with 1.0 mL of TRIzol (Thermo Fisher Scientific, Waltham, MA, USA), homogenized, and stored at -70 °C.

### 4.5. Quantitative PCR

Total RNA was extracted from the cells or organs using a TRIzol RNA purification kit (Thermo Fisher Scientific, Waltham, MA, USA), as recommended by the manufacturer, with subsequent DNase treatment (Thermo Fisher Scientific, Waltham, MA, USA). cDNA was synthesized from 2 μg of total RNA using the M-MLV reverse transcriptase (Evrogen, Moscow, Russia) with random primers. The obtained cDNA was amplified using a LightCycler 96 instrument (Roche, Basel, Switzerland). The reaction conditions were as follows: denaturation at 95 °C (3 min), followed by 40 cycles (95 °C, 15 s; 55–65 °C, 20 s; and 72 °C, 45 s). The reaction mixture qPCRmix-HS SYBR (Evrogen, Moscow, Russia) was used. As a reference gene, the 18S rRNA was used. Relative changes in the gene expression levels were determined using the 2^−ΔΔCt^ method [45]. The utilized primer sequences are given in Table S1.

### 4.6. Western Blot

An RIPA buffer (Thermo Fisher Scientific, Waltham, MA, USA) containing a mixture of protease inhibitors (Sigma-Aldrich, St. Louis, MO, USA) was used for cell and tissue lysis. The total protein concentration in the samples was determined using the BCA method [46]. An equal amount of protein (20 μg per sample) was separated by SDS-polyacrylamide gel electrophoresis (SDS-PAGE) and transferred to a PVDF membrane for protein blotting (Bio-Rad, Hercules, CA, USA). The membranes were blocked in 5% milk and incubated in 1% milk with rabbit anti-TRIM8 (1:750, ab155674, Abcam, Waltham, MA, USA), rabbit anti-TRIM14 (1:750, MBS9414054, MyBioSource, San Diego, CA, USA), rabbit anti-TRIM17 (1:750, CSB-PA897559LA01HU, CUSABIO, Houston, TX, USA), mouse β-Actin (1:6000, A5441, Sigma-Aldrich, St. Louis, MO, USA), or rabbit anti-GAPDH (1:1000; MA5-15738, Thermo Fisher Scientific, Waltham, MA, USA) primary antibodies at 4 °C overnight, washed 5 times for 5 min with TNT solution, and incubated in 1% milk with horseradish peroxidase (HRP)-conjugated secondary antibody (1:10,000; 31466, Thermo Fisher Scientific, Waltham, MA, USA; or 140777, Jackson ImmunoResearch, Cambridgeshire, UK) at RT for 2 h. The signal was recorded with an enhanced chemiluminescence reagent (No. 170-5061, Bio-Rad, Hercules, CA, USA) using the ChemiDoc MP Imaging System (Bio-Rad, Hercules, CA, USA).

### 4.7. Bioinformatic Analysis

The data used for analysis were obtained from GEO NCBI, accession numbers GSE121359 (row counts [17]) and GSE124007 (row sequencing data [18]). The reads from the GSE124007 project were aligned with the reference using the transcriptome index, built on the basis of the Mus musculus GRCm39 (release 109) reference genome with HISAT2 (v. 2.2.1) [47]. The obtained SAM files were converted into BAM format and sorted using Samtools (v. 1.10) [48]. Count matrices were created from the BAM files with HTSeq (v. 2.0.2) [49]. The count data from both datasets were ported to the R package DESeq2 (v. 1.38.3) [50] for downstream statistical and differential gene expression analysis.

### 4.8. Statistical Analysis

Statistical analysis was performed using GraphPad Prism 8.0 (GraphPad Software Inc., CA, USA). Statistical analysis of the PCR data and Western blot analysis was performed using a two-tailed unpaired *t*-test. A multi-factor ANOVA was used to estimate the differences between the expression profiles of *TRIM* genes (log2FCs) across groups of different pathogen types and times after infection. Pairwise comparisons of the expression profiles for different states of the model, grouped by the aforementioned factors, were conducted using Tukey’s range test. All calculations were made using the functions of the statistics package for R [51]. A *p* value of <0.05 was considered statistically significant.

## Figures and Tables

**Table 1:** *TRIM* genes whose transcription was increased in human cell lines or in mouse organs after infection compared to the controls (*p* < 0.05).

	*P. aeruginosa*				*Chlamydia* spp.					
	A549/PA	Mouse Lung/ PA	U937/PA	Mouse LN/PA	A549/CP	PC-3/CT	Mouse Lung/CM	U937/CP	U937/CT	Mouse LN/CM
A549/PA	16 ^1^, 17, 18, 19, 20, 21, 47, **52** ^2^, 63	16, 17, 18, 19, 20, 21	no	no	16, 18, 19, 20, 21, 47, 63	16, 17, 47	19, 20, 21	no	no	no
Mouse lung/PA	16, 17, 18, 19, 20, 21	1, 6, 8, **10**, 11, **12**, 13, 14, 15, 16, 17, 18, 19, 20, 21, 25, 26, 27, 30, 34, 56, **58**, 68	8, 14, 68	no	1, 8, 13, 14, 15, 16, 18, 19, 20, 21, 25, 27, 56, 68	16, 17, 27	6, 8, 11, 14, 15, 19, 20, 21, 26, 30, 34, 56, 68	8, 14, 68	8, 14, 26, 68	no
U937/PA	no	8, 14, 68	8, 14, 68, 74	no	8, 14, 68, 74	no	8, 14, 68	8, 14, 68	8, 14, 68, 74	no
Mouse LN/PA	no	no	no	no	no	no	no	no	no	no
A549/CP	16, 18, 19, 20, 21, 47, 63	1, 8, 13, 14, 15, 16, 18, 19, 20, 21, 25, 27, 56, 68	8, 14, 68, 74	no	1, **4**, **5**, 8, 9, 13, 14, 15, 16, 18, 19, 20, 21, **22**, **23**, 25, 27, **31**, **35**, 36, **39**, 47, **50**, **55**, 56, **61**, 63, **65**, 68, **69**, **71**, 74	16, 27, 47	8, 14, 15, 19, 20, 21, 56, 68	8, 14, 68	8, 14, 36, 68, 74	no
PC-3/CT	no	16, 17, 27	no	no	16, 27, 47	**3**, 16, 17, 27, **33**, **37**, 47	no	no	no	no
Mouse lung/CM	19, 20, 21	6, 8, 11, 14, 15, 19, 20, 21, 26, 30, 34, 56, 68	8, 14, 68	no	8, 14, 15, 19, 20, 21, 56, 68	no	6, 8, 11, 14, 15, 19, 20, 21, 26, 30, 34, 56, 68	8, 14, 68	8, 14, 26, 68	no
U937/CP	no	8, 14, 68	8, 14, 68	no	8, 14, 68	no	8, 14, 68	8, 14, 68	8, 14, 68	no
U937/CT	no	8, 14, 26, 68	8, 14, 68, 74	no	8, 14, 36, 68, 74	no	8, 14, 26, 68	8, 14, 68	8, 14, 26, 36, 68, 74	no
Mouse LN/CM	no	no	no	no	no	no	no	no	no	no

LN: lymph nodes; PA: *P. aeruginosa*; CP: *C. pneumoniae*; CT: *C. trachomatis*; CM: *C. muridarum*. ^1^ The *TRIM* genes, which are characteristic of each group, are highlighted in gray. ^2^ Unique genes for each group are in bold.

**Table 2:** *TRIM* genes whose transcription was decreased in human cell lines or in mouse organs after infection compared to the controls (*p* < 0.05).

	*P. aeruginosa*				*Chlamydia* spp.					
	**A549/PA**	**Mouse Lung/PA**	**U937/PA**	**Mouse LN/PA**	**A549/CP**	**PC-3/CT**	**Mouse Lung/CM**	**U937/CP**	**U937/CT**	**Mouse LN/CM**
A549/PA	7 ^1^	7	no	7	no	no	7	no	no	7
Mouse lung/PA	7	7, 63	no	7	no	no	7, 63	no	no	7
U937/PA	no	no	58, 59, **61** ^2^, 65, **66**, **71**	59, 65	no	no	65	58	65	65
Mouse LN/PA	7	7	59, 65	7, **12**, **13**, **14**, **17**, **21**, 26, 27, 28, 32, **37**, 38, 41, 46, 47, 56, **59**, 65, 72	no	no	7, 32, 65	no	65	7, 26, 27, 28, 32, 38, 41, 46, 47, 56, 65, 72
A549/CP	no	no	no	no	67	no	67	no	no	67
PC-3/CT	no	no	no	no	no	no	no	no	no	no
Mouse lung/CM	7	7, 63	65	7, 32, 65	67	no	**1**, 7, 32, **55**, 63, 65, 67	no	65	7, 32, 65, 67
U937/CP	no	no	58	no	no	no	no	58	no	no
U937/CT	no	no	65	65	no	no	65	no	65	65
Mouse LN/CM	7	7	65	7, 26, 27, 28, 32, 38, 41, 46, 47, 56, 65, 72	67	no	7, 32, 65, 67	no	65	**3**, 7, **8**, **18**, **23**, **24**, 26, 27, 28, 32, **33**, **35**, 38, **39**, 41, **45**, 46, 47, 56, 65, 67, 72

LN: lymph nodes; PA: *P. aeruginosa*; CP: *C. pneumoniae*; CT: *C. trachomatis*; CM: *C. muridarum*. ^1^ The *TRIM* genes, which are characteristic of each group, are highlighted in gray. ^2^ Unique genes for each group are in bold.

## Data Availability

The data presented in this study are available in this article or in the Supplementary Materials.

## Supplementary Materials

The supporting information can be downloaded at: https://www.mdpi.com/article/10.3390/ijms241713290/s1. References [15,16,17,18,52] are cited in the supplementary materials.
