# Supplementary material for: Identification of Key TRIM Genes Involved in Response to Pseudomonas aeruginosa or Chlamydia spp. Infections in Human Cell Lines and in Mouse Organs
Source: Int J Mol Sci. 2023 Aug 27;24(17):13290. doi: 10.3390/ijms241713290 (PMC10487655; doi:10.3390/ijms241713290)
Supplement: Supplementary file 1 [file ijms-24-13290-s001.zip › ijms-2530058-supplementary.pdf]

Table S1. Primers used in this study.

Human genes	Primer sequences (forward/reverse)	T, °C
18s RNA	**F**: CGGCTACCACATCCAAGGAA **R**: GCTGGAATTACCGCGGCT	60
IFNA	**F**: CCTGATGAATGCGGACTCCA **R**: TAGCAGGGGTGAGAGTCTTTG	63
IFNB	**F**: AGAAGGAGGACGCCGCATTG **R**: CCACTCTGACTATGGTCCAGGC	60
IFNG	**F**: ACTAGGCAGCCAACCTAAGC **R**: TTGGAAGCACCAGGCATGAA	60
TNFA	**F**: CTCCAGGCGGTGCTTGTT **R**: AGGCTTGTCACTCGGGGT	60
IL1B	**F**: GCTCGCCAGTGAAATGATGG **R**: GTCCTGGAAGGAGCACTTCAT	60
IL6	**F**: CCTTCCAAAGATGGCTGAAA **R**: CAGGGGTGGTTATTGCATCT	60
TRIM1	**F**: CGCCACGAGGCCTGTATAAT **R**: CTGGGGTGTGGCTCTTCTTT	60
TRIM2	**F**: AGGAGTGGCCGTTATGGAAC **R**: AGAAAGTGTGCAGACAGGGG	60
TRIM3	**F**: GCAAACGAAAGGACAACCC **R**: TGCTGTCTGCTACCACGATG [3]	55
TRIM4	**F**: ATGCTAAAGCGATTCCAAGTG **R**: CAAGAACTGGCTGATGCTGTAT [3]	60
TRIM5α	**F**: CGACGCTACTGGGTTGATGT **R**: ACTTTGAGAGCCCAGGATGC	60
TRIM6	**F**: GTGCGGGTCAGAGAGGATTC **R**: CCTGCCATTTGGTGTGATGC	60
TRIM7	**F**: GGCGAACCCTTCAAGCTCTA **R**: TCAGGCCTCTGTAATGTCCC	60
TRIM8	**F**: GACGGAGGATGTCAGCTTCA **R**: TCGTTCAGGAAGAGCTTGGA [3]	55
TRIM9	**F**: ATTCCGGGAGGTGTATGTGG **R**: CACCACCCGGTCATCATAGC	60
TRIM10	**F**: GGATGCAAGTCCTCCTGACTC **R**: TCCCGTTGCCTCAAGATGTC	55
TRIM11	**F**: CGTCTTCGCCGTTTGCT **R**: CCTCAGCTCCATAGGCACAA [3]	65
TRIM13	**F**: CCTTGTCATTGTCTTTGGTCCT **R**: ATTCTGCCACATTGTTCAGTACC [3]	60
TRIM14	**F**: CATTACAGACGCCATTGGACAT **R**: TGGTGAGGGGCTGGTTTTC	60
TRIM15	**F**: CAGCCAGCAAGTGAGCTTCTA **R**: CCAGAGTGATGACCCCTGAAT	55
TRIM16	**F**: GTGGCCACACCATAGTCTCC **R**: GCTTTGACCTCTGACACCGA	60
TRIM17	**F**: ACGCATTGTGCTGGAGTTTG **R**: CTGCTCAGGGGTTCCTTCAT [3]	55
TRIM18	**F**: AAAGCCACTCACCCGAATAA **R**: TTCTCATCCTCATGCTCCAA [3]	60
TRIM19	**F**: CGGAAGACTCAGATGCCGAA **R**: ACACGGCCTTGGAGTAGATG	60
TRIM20	**F**: CCACTCTGCTGGTCACCTACTAT **R**: GGAATCATCTGTGCCGTTTT [3]	60
TRIM21	**F**: GAAGCTCCAGGTGGCATTAG **R**: GCACAAACTCTGCGTGAATC [3]	60
TRIM22	**F**: TTATATCCAGATCGAGAGACAGAAG **R**: CATGACGTCAATCACATCCTGC	60
TRIM23	**F**: TGCCAGCATCCCTGTCACT **R**: TCCTAACGTAACGACCCGAAT [3]	60
TRIM24	**F**: CCCATCCAGCAACCTTCCAT **R**: TGCACTAGTAATCGTGGGGC	60
TRIM25	**F**: AGCATCAAAACTGCGAGGAAT **R**: GGGAGGTTTCTTGGATTTCTTT [3]	60
TRIM26	**F**: GAGGGAATTCCAGGGGAAGC **R**: GTGCAGGTAGGCACTCTTGT	60
TRIM27	**F**: CCAGAACCAGCTCGACCATT **R**: GGAGGCGATACTCATGCTCC	60
TRIM28	**F**: GCGGAAATGTGAGCGTGTA **R**: AGAAGGTGGAGTCGGTAGCC [3]	60
TRIM29	**F**: ATCGGCAACAAGCAGAAGG **R**: ATGATTCTTGTGCTCCTGGAAC [3]	65
TRIM31	**F**: TCGTCTGCTGGGAAAGTCAC **R**: TCACAAAACCAAGCCCGGAT	60
TRIM32	**F**: AATCGGCAGAATGAGCACCA **R**: CTTGCGACTACTGTCAGCCA	60
TRIM33	**F**: GCCCTGTCTTCACTCCTTCT **R**: GCATTCTTGGCGGCATACT [3]	60
TRIM34	**F**: TCAGATGTGGAGTGTCGGAGT **R**: TGATTCCACTCATGTCCTGC [3]	60
TRIM35	**F**: CATCGCCAAGCACAATCAG **R**: GCTCCTCCACTCTCAAGAACTC [3]	60
TRIM36	**F**: TCCAACTACTAACTTCAGACCCA **R**: CCAACTTACACAGATGGCAAAC [3]	60
TRIM37	**F**: TCAGCTGTATTAGGCGCTGG **R**: AAGTGCACACTGATGGCAGA	60
TRIM38	**F**: GCCAGGGCTACAAGGAAAAG **R**: GCTTCTGCTCCGTACATCTGTC [3]	60
TRIM39	**F**: TACCGCAGTCTCCGACCTAA **R**: AGCCTCCTGGTCCTCATAACA [3]	60
TRIM40	**F**: GCGGCCAGAATCCTTGACAT **R**: AAGTCACCAGCATTCTTCAGTG	65
TRIM41	**F**: GAGGTGGTGCAGGAGTACAAG **R**: TCCTCCTTGGCTTTCATCTTC [3]	60
TRIM42	**F**: TGCTATGTGCGTTTGCTGTC **R**: CGCTCTGAGGTGAAGATGAAC [3]	60
TRIM43	**F**: TTCCTCTGGAGGGGCAATGT **R**: CACTCCTTGCCACGATGTCT	60
TRIM44	**F**: TCCAGTCATTGGGGCTCAC **R**: TCCCGAGTTACTTTAGGGTCTG [3]	60
TRIM45	**F**: AAGACAGCCCAGTCAGAACAAT **R**: CAGAAGGTGCAGCAGTGAAAC [3]	60
TRIM46	**F**: TGCTTGAGAACCCCGACA **R**: TCGCTGGTCCTTGCTGATAG [3]	60
TRIM47	**F**: TGATGCTGAGCCCCAAGAC **R**: CCAGTAGTAGGTGCCTCGGT [3]	60
TRIM48	**F**: CTCAGGAGCACCGGTATCAC **R**: TGTCTCCAAAAGCCTTCCAGT	65
TRIM49	**F**: GGAATCTTACAGGTCTTTCAGGG **R**: GTTTATCTGCTCGGTTGACTTTG [3]	60
TRIM50	**F**: ATCGCCAAACTGGTGAACAA **R**: TGAAGACATCCGACTCATTGAC [3]	60
TRIM51	**F**: GCCTCAGTGGATTCAGAGTTGAT **R**: AGATGTGAAAGCCTGAGCCC	60
TRIM52	**F**: CTTTCGTCCCAACTTGCAGC **R**: AGTTGTTTCCAACTTTATTTCCTGG	60
TRIM54	**F**: ATCCTCTATGGCAGTCCCG **R**: CCTGGATGACTCCTGCTTGTA [3]	60
TRIM55	**F**: CCCAAGTCATTACCCGAACC **R**: TGCCTTTGATGCTTCCGAG [3]	60
TRIM56	**F**: AGGATGGTGGGAAAGACGG **R**: CTCCCTCTTCTCGGGTTGTC [3]	60

TRIM58	**F**: GCAGAAGTAAGGCTGTCACAAG **R**: AATCGCTCAGGGTTGTTGG [3]	60
TRIM59	**F**: CGTGTACTGCCATGCTCTCA **R**: TGTTCAGGGCAGGTGACAAT	60
TRIM60	**F**: ACCCCCAGCTCCGTAATTTG **R**: ATTCCTCAAGGGCTCAAGGC	60
TRIM61	**F**: AAGAAGCAAGAAGAGGAAGAGGC **R**: ACGGTGCATTGTATTCCTCCA	60
TRIM62	**F**: CAGTACACCATCTGGAAGTCCC **R**: CAAGTTGCCGTAAGCCACA [3]	60
TRIM63	**F**: GAGGATTCCCGTCGAGTGAC **R**: GTTGCTTGGCAGTCAAGAGG	60
TRIM64	**F**: AAAGCCCCAGCCAGTGAA **R**: GTGGGAGCACTGAGATGGTC [3]	60
TRIM65	**F**: CCCAAGCACAGTTTGTCCAC **R**: GCGACAGATAGAAGTGACGGTT [3]	60
TRIM66	**F**: TGCACAGAGGAACACCGAC **R**: CGGGACCACCATTCACTC [3]	65
TRIM67	**F**: AGGAAAACGGACTGGACTACG **R**: CATCCACAAGGGCATCACA [3]	60
TRIM68	**F**: AATGGGGCTGAAGGGTGA **R**: CCATACAATACTCTGTTTCGGG [3]	60
TRIM69	**F**: TGAGCATTCAGGCAAAGACG **R**: TGGTTGCCAGCACCTTCAT [3]	60
TRIM71	**F**: AAGAAAGCCCTGGAGGAACG **R**: GGGTGTGAACATGACTCGGT	60
TRIM72	**F**: GGAGGAGACAGTGCGTCAGTT **R**: GCTCCAGGTAAGAGTTCAGGC [3]	60
TRIM74	**F**: CGGACCCGAATCGTCAATGAG **R**: TCCGAACTGTTCCAGCACAC	60
TRIM75	**F**: GCCCATAGAGAAAGCTGCCA **R**: TGGAGAGAACTGCCTGTTGC	60

Mouse genes	Primer sequences (forward/reverse)	T, °C
18s RNA	**F**: GTAACCCGTTGAACCCCATT **R**: CCATCCAATCGGTAGTAGCG	60
IFNA	**F**: ATGGCTAGGCTCTGTGCTTTCCT **R**: AGGGCTCTCCAGACTTCTGCTCTG	60
IFNB	**F**: TGAATGGAAAGATCAACCTCACCTA **R**: CTCTTCTGCATCTTCTCCGTCA	63
IFNG	**F**: AACTCAAGTGGCATAGATGTGGA **R**: TGCTGTTGCTGAAGAAGGTAGTA	63
TNFA	**F**: CCCTCACACTCAGATCATCTTCT **R**: GCTACGACGTGGGCTACAG	60
IL1B	**F**: GCAACTGTTCCTGAACTCAACT **R**: ATCTTTTGGGGTCCGTCAACT	60
IL6	**F**: TCTGGGAAATCGTGGAAATGAG **R**: TCTCTGAAGGACTCTGGCTTTGTC	60
TRIM1	**F**: CAGTGGGAACCTTCTGGGAC **R**: CGCGGATCTTGAGGGTGAAA	60
TRIM2	**F**: AGCGACTCTGAAGAAGAGCAA **R**: GCACCTCTTGAGGACCAGTG	60
TRIM3	**F**: GGGGCCAAACAGAAGGTGTT **R**: TAGCAAAACCTCAGGGGCAG	55
TRIM4	**F**: ATGCTAAAGCGATTCCAAGTG **R**: CAAGAACTGGCTGATGCTGTAT	60
TRIM6	**F**: TCCAGGTATCAGCCACAGTG **R**: TAGGGACAAAGGGCAGAAGG	60
TRIM7	**F**: CTCTACTGCCAGGACGACG **R**: CTTCTTCAAGACCCTCAGCC	60
TRIM8	**F**: GACGGAGGATGTCAGCTTCA **R**: TCGTTCAGGAAGAGCTTGGA	59
TRIM9	**F**: ATGGAAGAGATGGAAGA **R**: CGCACGCCTGACATAAATTG	55
TRIM10	**F**: GCTGGCTTGGGGCTTTGTTTCA **R**: CACTCGAGGGTCTGCTCCCAAGGGTGCTTG	60
TRIM11	**F**: GCAGAAATGGCACGACGCCT **R**: GGAACAGCATTGCGTCCTCCA	60
TRIM12	**F**: AACGCTACTGGGTTCAGGTG **R**: CCCCCTGCGAAATAAAGGGT	60
TRIM13	**F**: AGGAGCAGCGGATGGCCTTCAACAT **R**: AGTGAACACACCTGTGTCTTGCGGC	60
TRIM14	**F**: CCCATTTGGAAGACGCCG **R**: AGGGTGGCTCAGCTCCG	60
TRIM15	**F**: CCTGAGCGAGACCTACTGTGA **R**: AGAGCTTCTAACCGACTCCTG	60
TRIM16	**F**: GTGTGACTTCTGTCTTGGGGC **R**: GGTCCACCTCTTTGTTCCTGC	60
TRIM17	**F**: TTCCAAGAGGATGTGATGCCG **R**: TGTCCTTCCGACTCACGTTG	65
TRIM18	**F**: AACACAGAACTGGAGACTCTTT **R**: CTTCTGTCAGTTTGGCTTCTTG	60
TRIM19	**F**: AGAGGAACCCTCCGAAGACT **R**: ATTCCTCCTGTATGGCTTGC	60
TRIM20	**F**: TGAAATGGCATTCAGCGCTTC **R**: CCAAGGTGCTCCCAATGAATG	60
TRIM21	**F**: GTCGAGGATTCACGCAGAGT **R**: CCAGGATCCACTCCTTTCCA	60
TRIM23	**F**: CATAGGCGTGTGCCTCTAGC **R**: GGTTCCAGTACTGAATGCTTGTG	60
TRIM24	**F**: GGTCCTTCGCCATTCGCC **R**: TCTCAGCACACTCTTGACTGC	60
TRIM25	**F**: CAACTTCCCCTGATGCCGC **R**: CCTCCCAGTAGTGAATGCCG	60
TRIM26	**F**: TGCAGGATGTCTGCACTACAC **R**: CAGGGTGACGCTTACTGTCTT	60
TRIM27	**F**: GCAAATCCAGAACCGACTGG **R**: GAGTCAGGCTCAAAAGCTCAG	60
TRIM28	**F**: AGGATGCTAACCAGTGCTGC **R**: ACTGATGGTCCTTGTGAGCG	60
TRIM29	**F**: ACCTCCTGTGACCTTTGCTG **R**: GGTAGCTATTCCTGCGGACT	60
TRIM30	**F**: ACCTTAGAAATGCTGCAGGGTG **R**: TCCCAGGACACCCAAATGGTA	60
TRIM31	**F**: GCGCCAGAGACTAGATGAGG **R**: TGAAATTCCTTACCCCTGCTCA	60
TRIM32	**F**: TCTCCATCGGTTCTGTTGGC **R**: GGCCCTCTCGAATAAGGACG	60
TRIM33	**F**: CTGTGGACCAAAGGAAATGTGA **R**: TCTTGTGTGTCTGCATAAACTTGA	60
TRIM34	**F**: GGGCCTACTGGGACAACTTC **R**: TTTGGCTGACCTTTGCGTTG	60
TRIM35	**F**: CCAACCACACGCTCAACAAC **R**: GTTCTTACACTTGGCCCGGA	60
TRIM36	**F**: TGCCAAGGGCAAGGTGACTA **R**: TCGGGGTCAGTGAATTACGC	60
TRIM37	**F**: GGTCCTCCAGGTAGTAGCCA **R**: CCTGCTCCTCGTTCTCAGTG	60
TRIM38	**F**: CACTTTGAGCAGGTGTGAGG **R**: GCTGACTTGGTGACGTCGTA	55
TRIM39	**F**: TCTTAAACAGCTAATTGCGGATG **R**: GTTGTAAAAGGACAGTGTGCCA	60

TRIM40	**F**: ATGGGCTCTCTTGACAAGGAC **R**: ACTGAAGCCTTATCCATGTGC	60
TRIM41	**F**: CGAATCCAGGAGCCACAAAC **R**: TCCACATGCCCTTGTAGCTT	60
TRIM42	**F**: CCCAGGCCCAATTGTCATCT **R**: TGGTGTTACCACCTTGCAGA	55
TRIM44	**F**: GTCCAGTCATTGGGGCTCAC **R**: TCCACCGGAATCTTTGCTTCTC	60
TRIM45	**F**: CCAGGCTCATAGGCGACAG **R**: CTACACAGTCCCGACACACC	60
TRIM46	**F**: ATGCAGACCTTCACTTCCATCA **R**: GGGCACAGCAGTTCCTTCTC	60
TRIM47	**F**: GTGGAGCGTGTGTGCCTTT **R**: GTTCATCCATGCGGTCCTCC	60
TRIM50	**F**: GCGCTTCGACTATAGCACCT **R**: GTAAGTAGACGCCGATGCGA	60
TRIM52	**F**: AGCCCTATGCAGTCACTTCG **R**: TTTGCATTGCCCTGATGCTG	60
TRIM54	**F**: AGCTGATCAACAAGGTCGGG **R**: CTCCCCATCCAAAGCCATGT	55
TRIM55	**F**: CAAAGCCGGAAAACCAGCTC **R**: GCCTCAAATCCAATCTGAGAGG	65
TRIM56	**F**: TACAGAGCCGGGTGGTATGA **R**: GCAGGGGTGATCGATATGGG	60
TRIM58	**F**: GTTCTGCGAGAAGTCGGACA **R**: GCTCGGAGCATCCTCTGGTA	65
TRIM59	**F**: CATATGGAGGCCTCTGCGAA **R**: GGTGACAACATCTGGGTGGT	60
TRIM60	**F**: CTGTGTGAGGGATCGGGATG **R**: GGCGCTCTCTAGGACTTTCC	60
TRIM61	**F**: GGTATGCCGAACGGTTCTCT **R**: GTAAGGCCAAAATGTGCCCC	55
TRIM62	**F**: ACGATGCCTTCGAGGAGTT **R**: CTGTCCTGAAGGGCCTGA	60
TRIM63	**F**: GGGCTACCTTCCTCTCAAGTG **R**: TTTACCCTCTGTGGTCACGC	60
TRIM65	**F**: AGATGCTGGCCCTTTGGAGA **R**: AGTGCAAATACTGGTTGGCG	60
TRIM66	**F**: AGCCACACTGCTCTACAGATG **R**: CAGGAGATGAGCTTACCTGGG	55
TRIM67	**F**: TGGAGCCCAAAGTATCTGCG **R**: GGTGGCAATTTCATCTGGACG	60
TRIM68	**F**: CGCTGGATGCTGCAGGGTAT **R**: TCTGGATCTAGACGCACATCCACT	60
TRIM69	**F**: ATTCTCTCTCCAGGCCCATCT **R**: AGCCAGAATCCTTGCTCTGG	55
TRIM71	**F**: GATCGGGAGTGTGAGCTGTT **R**: TGCAGGAACAGAGACTTCGC	60
TRIM72	**F**: CCCGACGTACTTACGCCAAT **R**: CATTCTTGCCCTTGTCGTGC	60
TRIM75	**F**: GACCTGAAGACAGCCCATCC **R**: AGCTCCTGAGACGTTGAAGC	60

Table S2. Analysis of *TRIMs* expression in human cell lines (this work) after bacterial infection in comparison to macrophages and lymphocytes upon different infections and various stimuli [15,16]. Red, up-regulation of gene expression, blue, down-regulation of gene expression.

	Our data						Jiang et al., 2017 [15]												Carthagena et al., 2009 [16]	
	PC3	U937			A549		THP1-derived macrophages										MDM	PBL	MDM	PBL
TRIM	*CT*	*CT*	*CP*	*PA*	*CP*	*PA*	*TLR1/2*	*TLR2*	*TLR3*	*TLR4*	*TLR5*	*TLR6/2*	*TLR7*	*TLR8*	*TLR9*	*TNF*	IFN I		IFN II	
1																				
2																				
3																				
4																				
5																				
6	Ne	Ne	Ne	Ne																
7		Ne	Ne	Ne																
8																				
9																				
10																				
11																				
13							*	*	*	*	*	*		*	*					
14																				
15																				
16																				
17																				
18																				
19																				
20	Ne																			
21																				
22	Ne																			
23																				
24																				
25																				
26																				
27																				
28																				
29	Ne																			
31																				
32																				
33																				
34																				
35																				
36																				
37																				
38																				
39																				
41																				
44																				
45																				
46																				
47		Ne	Ne	Ne																
48																				
49																				
50																				
52																				
54		Ne	Ne	Ne																
55		Ne	Ne	Ne																
56																				
58																				
59																				
60																				
61																				
62																				
63																				

65
66
67
68
69
71
72
74

H.s., Homo sapiens, CT, Chlamydia trachomatis, CP, Chlamydia pneumoniae, PA, Pseudomonas aeruginosa, CM, Chlamydia muridarum, TLR, Toll-like receptor, TNF, tumor necrosis factor, MDM, monocyte derived macrophages, PBL, peripheral blood lymphocytes, Nd, not determined, Ne, no expression.

THP1 cells, a human monocyte cell line. MDM or PBL were cultivated in the presence or absence of IFN I (Universal type I IFN is a hybrid alpha interferon, constructed from recombinant human IFN-α A and human IFN-α D) or IFN II (IFNγ) [16].

* [52]

Table S3. Comparison of *TRIM* expression in mouse organs after bacterial infections (qPCR from this work, RNA-seq data from [17] and [18]), in human macrophages and lymphocytes upon different infections and various stimuli [15,16]. Red, up-regulation of gene expression, p<0.05, blue, down-regulation of gene expression, p<0.05. More light colors demonstrate tendency without significance.

	M.m. lung	*M.m.* lung	M.m. lymph nodes	*M.m.* lymph nodes	*H.s.* macrophages [15]	*H.s.* MDM or PBL [16]	*H.s.* MDM or PBL [16]	*M.m.* lung [18]	*M.m.* lung [17]
TRIM	*CM*	*PA*	*CM*	*PA*	TLR stimulation	IFN I	IFN II	*CM, 7 days p.i.*	*PA, 24 h p.i.*
1									
2									
3									
6					3,4				
7					2,5				
8									
9									
10					1-5,7-9, TNF				
11									
12								Trim12c	
13					1-5,8,9*				
14					3,4				
15					2-5,8,9,TNF				
16									
17									
18					2,6				
19					3,4				
20			Ne	Ne	3,4				
21					3,4				
23									
24									
25					3,4				
26					3				
27									
28									
29					1-5,7-9,TNF				
30									
31			Ne	Ne	2,3,4,5,8,TNF				
32									
33									
34					3,4				
35									
37					1				
38					3				
39							8		
41					4				
44									
45									
46									
47									
50					2,4,5,8,TNF				
52									
54									
55					1,6,7,TNF				
56					3,4				
58			Ne	Ne	2				
59					3,4				
60					2-5, 7-9, TNF				
61					1-5,7-9,TNF				
62									
63					2,5				

65					2				
67					2,5,TNF				
68									
69					2,3,4				
71			Ne	Ne	2,4,5,8, TNF				
72					2,8,TNF				

H.s., *Homo sapiens*, *M.m.*, *Musculus*, *PA*, *Pseudomonas aeruginosa*, *CM*, *Chlamydia muridarum*, TLR, Toll-like receptor, TNF, tumor necrosis factor, MDM, monocyte derived macrophages, PBL, peripheral blood lymphocytes, Nd, not determined, Ne, no expression.

* [52]
